# The effectiveness and safety of electromyography biofeedback therapy for motor dysfunction of children with cerebral palsy: A protocol for systematic review and meta-analysis

**DOI:** 10.1097/MD.0000000000016786

**Published:** 2019-08-16

**Authors:** Ming-Xing He, Cong-Jie Lei, Dong-Ling Zhong, Qi-Cen Liu, Hong Zhang, Yi-Jie Huang, Yu-Xi Li, Xiao-Bo Liu, Juan Li, Rong-Jiang Jin, Li Wan

**Affiliations:** a School of Health Preservation and Rehabilitation, Chengdu University of Traditional Chinese Medicine, Sichuan; b Acupuncture and Rehabilitation Department, Zigong City Hospital of Traditional Chinese Medicine, Zigong; c School of Acupuncture-Moxibustion and Tuina, The Third Affiliated Hospital, Chengdu University of Traditional Chinese Medicine; d School of Chengdu Sports Institute, Chengdu, Sichuan, China.

**Keywords:** cerebral palsy, effectiveness, EMG biofeedback therapy, meta-analysis, safety, systematic review

## Abstract

**Introduction:**

To investigate the effectiveness and safety of electromyography (EMG) biofeedback therapy in improving motor dysfunction among children with cerebral palsy (CP).

**Methods and analysis:**

The following databases will be searched: PubMed, EMBASE, ScienceDirect, the Cochrane Library, China National Knowledge infrastructure (CNKI), Technology Periodical Database (VIP), WanFang Data and China Biology Medicine (CBM) from inception to June 2019. All relevant randomized controlled trials (RCTs) utilizing EMG biofeedback therapy for CP will be included. The main outcome is the Gross Motor Function Measure (GMFM). Additional outcomes such as the Modified Ashworth Scale (MAS), Integral Electromyogram (iEMG), Composite Spasticity Scale (CSS), passive range of motion (PROM) or other related outcomes will be included, adverse effects of EMG biofeedback therapy and comparators will also be included. Two reviewers will screen studies, extract data and assess quality independently. Review Manager 5.3 will be used to assess the risk of bias, data synthesis, and subgroup analysis.

**Ethics and dissemination:**

This systematic review does not require formal ethical approval because all data will be analyzed anonymously. Results will provide a general overview and evidence concerning the effectiveness and safety of EMG biofeedback therapy for children with CP. The findings of this systematic review will be disseminated through peer-reviewed publications or conference presentations.

## Abstract 2

Supplemental Digital Content is available in the text

## 1 Introduction

Cerebral palsy (CP) is a group of persistent central and posture developmental disorders, activity-restricted syndromes caused by non-progressive brain damage in developing fetuses or infants, ^[1,2]^ Its main manifestations are central dyskinesia and abnormal posture. The prevalence of the disease is about 1.5%_0_∼4.0%_0_, affecting 2 per 1000 live births globally,^[3]^ the incidence rate in China is about 1.8%_0_∼6.0%_0_. With the increase of the survival rate of low birth weight children, the incidence of CP is a rising trend in recent years.^[4]^The motor disorders of CP are often accompanied by disturbances of sensation, cognition, communication,^[5,6]^ impacting children's motor control, intellectual function, ability to perform daily activities and participation in society.^[7]^ CP is one of the main causes of disability in children's motor dysfunction, which is significantly related to quality of life in children,^[8,9]^ resulting in huge economic and psychological burdens on families and society.^[10]^ At present, there are multiple therapies have been used to treat CP, such as surgical methods of selective posterior rhizotomy, exercise therapy of Bobath and Vojta, cerebral gangliosides, herbs, acupuncture, and other traditional Chinese therapies. However, these therapies are mostly passive, cannot mobilize the various information to actively promote exercise learning, which is important in CP treatment. Therefore, it is necessary to find a non-invasive, safe, active and effective treatment for CP.

Electromyography (EMG) biofeedback therapy is an active, conscious rehabilitation training,^[11]^ by collecting the active motor muscle contraction intensity of the child to guide the repeated active training and closed-loop stimulation training through various forms of feedback (“audio and vision” or “immersion”),^[12]^ thereby effectively reconstructing the brain nerve and promoting the recovery of the limbs.^[13,14]^ A large number of clinical studies have demonstrated that EMG biofeedback has a positive effect in improving the limb function of children with CP.^[15,16,17,18]^ It can also improve the ankle joint function, muscle strength and walking speed in children with CP.^[19,20,21,22]^ Studies have shown that EMG biofeedback therapy can inhibit muscle tension in the diaphragm.^[23,24]^ However, there is currently no relative systematic reviews (SRs) have been conducted to investigate the effectiveness and safety of EMG biofeedback therapy for CP. Therefore, our SR aims to evaluate the effectiveness and safety of EMG biofeedback therapy among children with CP. We will conduct this SR and meta-analysis in accordance with A Measurement Tool to Assess systematic Reviews 2.0 (AMSTAR 2.0) and Risk of Bias in Systematic Reviews (ROBIS).

## 2 Methods and analysis

### 2.1 Study registration

The protocol of this SR has been registered on PROSPERO (CRD42019133097, http://www.crd.york.ac.uk/PROSPERO). This protocol is reported according to Preferred Reporting Items for Systematic Reviews and Meta-Analyses Protocols (PRISMA-P) statement guidelines.

### 2.2 Ethical considerations

Since this protocol is based on published studies, ethical approval and patient consent are not needed.

### 2.3 Inclusion criteria

#### 2.3.1 Type of studies

We will only include clinical randomized controlled trials (RCTs) using EMG biofeedback therapy to treat CP. We will include RCTs published in English or Chinese. There are no restrictions on publication status.

#### 2.3.2 Type of participants

Patients with CP who meet the diagnostic and classification criteria established by the 2004 National Symposium on Pediatric Cerebral Palsy and Diagnostic criteria of the 9th National Conference on Pediatric Cerebral Palsy Rehabilitation^[6]^ will be included. There are no restrictions on gender, race or nation.

#### 2.3.3 Type of interventions

RCTs that used EMG biofeedback therapy for CP will be included, and the duration is at least 4 weeks.

#### 2.3.4 Type of comparators

The comparative intervention could be conventional rehabilitation or usual care.

#### 2.3.5 Outcome measurements

The main outcome will be the Gross Motor Function Measure (GMFM); Additional outcomes will include the Modified Ashworth Scale (MAS), Integral Electromyogram (iEMG), Compopsite Spasticity Scale (CSS), passive range of motion (PROM), or other related outcomes. Adverse events such as overstrain for treatment or organ injury will also be taken into account as safety measurement.

### 2.4 Exclusion criteria

① Non-RCTs, such as case-control studies, cohort studies, cross-over studies, and reviews; ② Animal experiments and subjects including non-cerebral palsy cases; ③ Biofeedback therapy combined with other treatments (except conventional rehabilitation or usual care); ④ Duplicate or data cannot be extracted; ⑤ Full text cannot be obtained through various approaches.

### 2.5 Database and search

The following databases will be searched: PubMed, EMBASE, ScienceDirect, the Cochrane Library, China National Knowledge Infrastructure (CNKI), Technology Periodical Database (VIP), WanFang Data and China Biology Medicine (CBM) from inception to June 2019, using the key words of EMG biofeedback therapy, cerebral palsy, spasticity, muscle tension, motor function and RCTs. We will search the grey literature, and reference lists of identified articles will be checked to avoid missing eligible trials. We have developed the PubMed search strategy (see Appendix 1) based on guidance from the Cochrane handbook and will apply similar strategies for other electronic databases.

### 2.6 Studies selection

**Figure 1:**
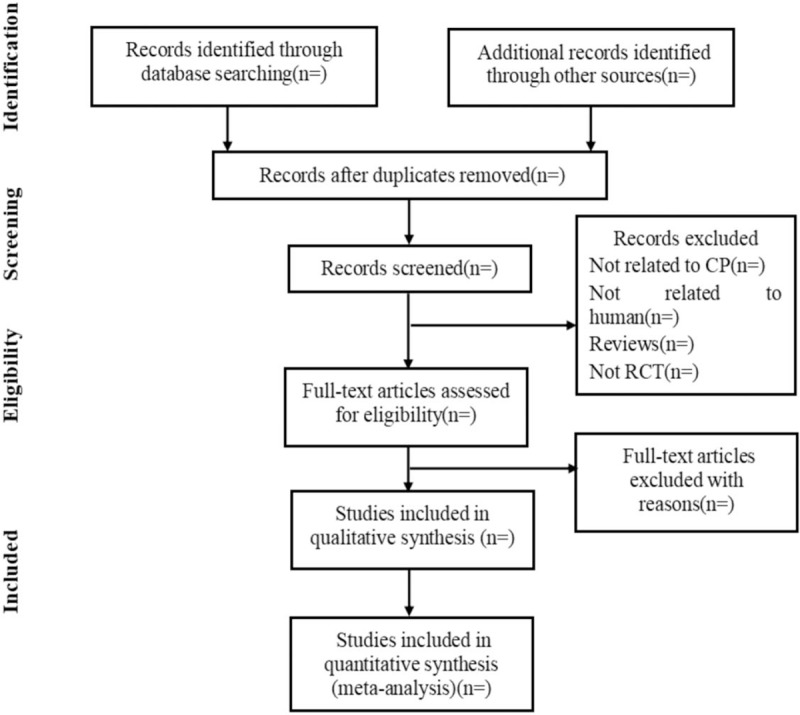
Flow chart of the study selection process.

All records will be managed with Endnote X8. Duplicates will be removed before screening. Two reviewers (MXH and XCL) will independently screen the titles and abstracts for potentially relevant studies. The 2 reviewers will then independently read the full texts based on the predetermined eligibility criteria. In case of unclear information or missing data, we will contact the original authors. Disagreements will be resolved by discussion and consultation with an experienced reviewer (JRJ). Details of the entire selection procedure will be shown in a flow chart (see Fig. 1).

### 2.7 Data extraction

Two reviewers (YXL and XBL) will independently extract data with a pre-designed data extraction form, in which study characteristics (first author, publication year and country, etc), participant characteristics (sample size, age, gender, health status, disease duration, etc), intervention characteristics (interventions, comparisons, duration of intervention, frequency of intervention, etc.), outcomes (main conclusions, adverse effect, etc) will be included. The original authors will be contacted in case of missing data. If the included RCTs involve multiple subgroup studies, only the experimental and control groups that are consistent with the objectives of this SR will be extracted. As for discrepancy, 2 reviewers will resolve through team discussion.

### 2.8 Assessment of risk of bias

Risk of bias will be assessed by 2 independent authors (CJL and DLZ) using the Cochrane risk of bias tool (www.cochrane-handbook.org), including the following items: random sequence generation, allocation concealment, blind subjects and therapists, blind assessors, incomplete outcome data, selective outcome reporting, and other bias. The risk of bias is categorized as low (meet all criteria)/unclear (trials with insufficient information to judge)/high risk (meet none of the criteria) of bias. In case of disagreements, a third reviewer (JL) will be involved.

### 2.9 Data analysis

The relative risk (RR) will be used to analyze dichotomous outcomes. The mean difference (MD) will be used to analyze continuous outcomes with the same unit. Otherwise, the standardized mean difference (SMD) will be used. The uncertainty will be expressed with 95% confidence intervals (95% CI). We will check the results of the χ^2^ test to assess the heterogeneity of included studies and the I^2^ statistic to quantify inconsistency. An I^2^ value of 50% or higher indicates the presence of substantial heterogeneity. If there is a low level of heterogeneity among included studies, we will synthesize the results with a meta-analysis. In case of substantial heterogeneity, we will perform a systematic synthesis instead. Results will be described qualitatively in the text when meta-analysis is not possible.

#### 2.9.1 Subgroup analysis

We plan to carry out subgroup analysis if sufficient comparable studies are identified. We intend to stratify the results by age, sex, and duration of EMG biofeedback therapy. We will also focus on subgroup analyses of comparison between EMG biofeedback therapy and other rehabilitation therapies.

#### 2.9.2 Sensitivity analysis

To ensure the robustness of evidence, we will perform sensitivity analysis to assess the impact of studies with a high risk of bias.

#### 2.9.3 Publication bias

We will assess reporting bias and will perform funnel plots to assess reporting bias if sufficient studies are included. We will try to explore possible interpretations other than publication bias and language bias if funnel plots are asymmetric.

#### 2.9.4 Dealing with missing data

We will contact the original authors in case of missing data. If there were no reply, we will only analyze the available data and address the potential impact of these missing data on the results of the review in the discussion section.

### 2.10 Grading of recommendations assessment, development, and evaluation (GRADE)

We will evaluate the quality of evidence of each outcome with the GRADE system. The quality of the index will be evaluated from the following 5 aspects: limitations, inconsistency, indirectness, imprecision, and publication bias.^[25]^ The quality of evidence will be graded as “high”, “moderate”, “low”, or “very low” in accordance with the GRADE rating standards. The results of GRADE including evidence profile (EP) and summary of finding table (SoF) will be generated using GRADE pro software.

### 2.11 Ethics and dissemination

This SR does not require formal ethical approval because all data used will be anonymous with no concerns regarding privacy. Results will provide a general overview and evidence concerning the effectiveness and safety of EMG biofeedback therapy for children with CP. Findings will be disseminated through peer-reviewed publications.

## 3 Discussion

EMG biofeedback therapy combines EMG with neuromuscular electrical stimulation (NMES). By measuring EMG, small neurological signals can be detected during exercise. When the dynamic EMG threshold is reached, an electrical stimulus is generated, the weak EMG signal generated by the active conscious muscle contraction of the child is amplified and then output, stimulating the corresponding muscle to induce significant muscle contraction movement. By completing the closed-loop stimulation mode and repeated active exercise training, the child gradually controls the muscles through feedback signals, which provides a strong guarantee for the normal movement of the limbs.^[26]^ EMG biofeedback therapy is effective for children with CP in improving lower limb motor function,^[27,28]^ gait speed,^[22,29]^ neuromuscular control and motor coordination,^[30]^ which has been widely used in clinical practice. However, the present researches on the efficacy of CP still unclear. Thus, it is necessary to conduct a SR to investigate the effectiveness and safety of EMG biofeedback therapy of children with CP. Therefore, we will conduct a SR and meta-analysis to assess the effectiveness and safety of EMG biofeedback therapy in children with CP, hoping our results may help clinicians and patients making decisions regarding the practice of EMG biofeedback therapy of CP.

## 4 Strengths and limitations

This SR will assess the effectiveness and safety of EMG biofeedback therapy among children with CP, and provide evidence on therapeutic effect of EMG biofeedback therapy for CP based on existing clinical researches. However, there are still some potential limitations. The proposed SR is only a comprehensive quantitative analysis of the existing literature results, and cannot replace large-scale, multi-center RCT. Besides, language bias may exist since we will only include studies published in English and Chinese, some studies in other languages will not be included, so relevant information may be missed.

## Author contributions

**Conceptualization:** Juan Li, Rongjiang Jin.

**Methodology:** Li Wan,Yuxi Li, Xiaobo Liu

**Writing – original draft:** Mingxing He, Congjie Lei, Dongling Zhong

**Writing – review & editing:** Qicen Liu, Hong Zhang, Yijie Huang.

## Supplementary Material

Supplemental Digital Content
