# Supplementary material for: The effectiveness and safety of electromyography biofeedback therapy for motor dysfunction of children with cerebral palsy: A protocol for systematic review and meta-analysis
Source: Medicine (Baltimore). 2019 Aug 16;98(33):e16786. doi: 10.1097/MD.0000000000016786 (PMC6831207; doi:10.1097/MD.0000000000016786)
Supplement: Supplemental Digital Content [file medi-98-e16786-s001.doc]

**Appendix 1 Search strategy for Pubmed**

#1　Neurofeedback[Mesh]

#2　Neurofeedback[Title/Abstract] OR Electromyography Feedback[Title/Abstract] OR EMG Feedback[Title/Abstract] OR EMG biofeedback [Title/Abstract] OR biofeedback[Title/Abstract] OR electromyographic feedback[Title/Abstract]

#3　#1 OR #2

#4　Cerebral Palsy[Mesh]

#5　Cerebral Palsy[Title/Abstract]) OR Cerebral Pals*[Title/Abstract]) OR cere-bral palsy[Title/Abstract] or CP[Title/Abstract]

#6　#4 OR #5

#7　Motion[Mesh]

#8　Motion[Title/Abstract] OR Motion*[Title/Abstract] OR motor function [Title/Abstract] OR movement function[Title/Abstract]

#9　#7 OR #8

#10　Randomized Controlled Trial[Publication Type]

#11　Randomized Controlled Trials[Title/Abstract] OR Clinical Trials, Randomized [Title/Abstract] OR Trials, Randomized Clinical[Title/Abstract]OR Randomized [Title/Abstract]

#12　#10 OR #11

#13　#3 AND #6 AND # 9 AND #12
